# Workplace Ostracism Seen through the Lens of Power

**DOI:** 10.3389/fpsyg.2017.01528

**Published:** 2017-09-05

**Authors:** John Fiset, Raghid Al Hajj, John G. Vongas

**Affiliations:** ^1^Faculty of Business Administration, Memorial University of Newfoundland, St. John’s NL, Canada; ^2^John Molson School of Business, Concordia University, Montreal QC, Canada; ^3^School of Business, Ithaca College, Ithaca NY, United States

**Keywords:** workplace ostracism, external social support, hierarchical status, approach/inhibition theory of power, organizational citizenship behavior, interpersonal deviance, turnover intention

## Abstract

Drawing on approach/inhibition theory of power, we investigated two factors that influence the manner by which victims react to workplace ostracism: the hierarchical status of the ostracizer and the level of an ostracizee’s external social support including family, friends, and significant others. Across an experimental vignette study (Study 1) and a field study (Study 2), we found support for a three-way interaction with felt ostracism, ostracizee external social support, and ostracizer status influencing victims’ organizational citizenship behavior and deviance directed toward other individuals. In addition, felt ostracism and ostracizee external social support interacted to predict turnover intentions. Overall, victims who were ostracized by a legitimate higher-status authority (e.g., manager) and whose external social support network was limited experienced the most negative outcomes across both studies. Our findings suggest that contextual factors both inside and outside the organization jointly impact the way in which individuals react to perceived workplace ostracism. Implications and future research directions are discussed.

## Workplace Ostracism: A Primer

Being isolated by others and excluded from group interactions at work is a painful experience. In the United States alone, most employees have reported experiencing some form of social exclusion (Fox and Stallworth, 2005). Workplace ostracism takes place whenever an individual or group, the ‘ostracizer,’ neglects to take actions that engage another employee, the ‘ostracizee,’ when it is customary and suitable to do so (Robinson et al., 2013). Although considered by many as a counter-normative behavior, ostracism is qualitatively different from more active forms of incivility such as sexual harassment, bullying, and supervisory abuse in two principal ways (Ferris et al., 2013; Robinson et al., 2013). First, ostracism is low in behavioral intensity. For example, excluding a subordinate from a group interaction would be a subtle gesture compared to publically reprimanding him or her for failing to meet a deadline. Second, ostracism is fraught with ambiguity. Perpetrators can easily justify their behavior as being benign, a mere oversight with no associated malevolence. If a victim were told that he or she would be ostracized due to a specific transgression, the act of exclusion would then represent an active form of punishment in which both the determinant and consequence are specified. However, even if ostracism is a subtle form of mistreatment, growing evidence has demonstrated that being denied social connection either by an individual or a group leads to harmful outcomes for the victim (Williams, 1997; Eisenberger, 2015; O’Reilly et al., 2015).

Theoretical work in this domain has noted that such outcomes originate from thwarting a person’s basic psychological needs of control, self-esteem, meaningfulness, and belongingness (Williams, 1997). Of these four, the need to belong has received the most empirical attention in its ability to clarify what people do following social exclusion (Baumeister et al., 2007; Stenseng et al., 2014). Indeed, victims seek to fulfill belongingness needs by pursuing a variety of strategies. For example, whereas some ostracizees may attempt to elevate their status within the rejecting group through ingratiation and extra effort, others may opt to react aggressively to induce acknowledgment and retribution (Baumeister and Leary, 1995). Still, there are others who might avoid the ostracizer and seek to replenish their need to belong from external sources. It may even be possible that individuals pursue combined strategies in order to fortify threatened needs. Whichever path is taken, each of these strategies is consistent with Williams’s (2007) classification of ostracism reactions that include affiliation (‘tend-and-befriend’), retaliation (‘fight’), and avoidance (‘flight’). Within this framework, we examine outcomes that can be conceptualized as manifestations of each of these three reactions. First, following perceptions of being ostracized by a single person at work, we analyze ostracizees’ organizational citizenship behavior directed at individuals as representing a tend-and-befriend strategy. Second, we explore the extent to which ostracizees engage in interpersonal deviance that corresponds to a fight strategy. Lastly, we investigate ostracizees’ turnover intention as representing a flight response.

Although studies have examined the effect of individual differences on reactions to ostracism (Williams and Govan, 2005; Nezlek et al., 2012), less is known about the role played by contextual factors. The fact that an ostracizee could engage in various responses such as flattering the source (Williams, 2007), complying to reduce future ostracism (Carter-Sowell et al., 2008), or securing alternative sources of belonging (Aydin et al., 2010) suggests the existence of potential moderators that may influence individual reactions to ostracism. Here, we explore two boundary conditions, namely the ostracizer’s hierarchical status and the ostracizee’s perceived external social support that we believe jointly influence the directionality of employee reactions to workplace ostracism. Before doing so, however, we explain why these two moderators deserve particular attention.

## Why Ostracizer Status Matters

It is reasonable to assume that a person’s reaction to being ostracized could depend on the ostracism source. Being shunned by an eminent peer, for example, could weigh differently on how one copes with the rejection compared to being spurned by someone having a lower social position. However, what is it about the ostracizer’s status *per se* that merits scrutiny when considering an ostracizee’s experience and subsequent behavior? Scholars broadly define social status as the deference that some people receive from others with the result being that those who are respected will be given control over coveted resources (Parsons, 1940; Magee and Galinsky, 2008). Perceptions of hierarchy appear to be rooted in evolution and, even when deliberate attempts are made to curtail stratification through social engineering, status differences persist both within and between groups (Sidanius and Pratto, 1999). Thus, attaining and maintaining status within one’s social group remains a fundamental human pursuit (Anderson et al., 2001).

Status is thought to play an important role in reactions to workplace ostracism (Hitlan et al., 2006; Bozin and Yoder, 2008). In a recent review, Robinson et al. (2013) argued that the ostracizer’s social value affects how threatening an ostracism episode is to the victim and, consequently, how the latter might respond. For example, whenever an ostracizing party wields considerable influence, the ostracizee might be motivated to act prosocially in order to be reinstated in the group. Understanding how status relates to ostracism, however, requires an appreciation of the meaning of status. Whereas Nicholson and de Waal-Andrews (2005) likened status to an objective measure of success, along with material possessions, reputation, knowledge, and skills, Magee and Galinsky (2008, p. 354) defined it as “an implicit or explicit rank order of individuals or groups on a valued social dimension.” Hierarchical status therefore involves an awareness that people have regarding not only their location within a social ranking system but also that of others. Studies have shown that people have good reason to maintain relationships with influential group members as they are often seen as an embodiment of the organization itself (Eisenberger et al., 2010). For example, positive leader-member exchanges have been linked to followers’ increased wellbeing and performance (Epitropaki and Martin, 1999; Markham et al., 2010). For these reasons, we focus on the hierarchical status of ostracizers; that is, the status they possess by virtue of their legitimate organizational role. This type of status is both objective and conspicuous unlike other types that may be more subjective (e.g., prestige; Benoit-Smullyan, 1944). Hierarchical status is therefore reliable to measure and resistant to perceptual biases. However, the consequences that ensue whenever high-status members ostracize lower-status individuals remain elusive and one plausible response for ostracizees is to turn to their external network for support.

## Ostracizee Reliance On External Social Support

Social support has been a foundational concept in the study of occupational stress and wellbeing, with studies showing that high levels of communal support improve mental health and the ability to cope with traumatic events (Taylor, 1995; Lakey and Orehek, 2011). However, the lack of agreement on the meaning of social support plagues knowledge development in this domain. This misunderstanding has been partly attributed to overlooking the existence of distinct types of support and the theoretical perspectives that describe how support influences individual outcomes (Barrera, 1986; Zimet et al., 1988; Lakey and Cohen, 2000). One conceptualization of social support is that it represents the resources and structures of one’s networks (Greenhaus and Parasuraman, 1994; Marcinkus et al., 2007; Wills and Ainette, 2012). These resources serve different purposes and come in many forms, including emotional, informational, economic, and companionship support (Cohen and Wills, 1985; Wills, 1985; Scott et al., 2014). Moreover, these resources comprise two distinct types, perceived and enacted, with the former being subjective judgments of their existence and the latter representing support in concrete and observable terms (Barrera, 1986; Dunkel-Schetter and Bennett, 1990; Lakey and Drew, 1997). We focus on perceived external social support, which is more readily assessed as it does not rely on particularly taxing retrospective observations of support received by others. For example, while it might be relatively easy for one to recall and record enacted support in the form of behaviors (e.g., receiving a loan from a family member), it becomes far more difficult to objectively assess enacted support in other forms (e.g., receiving emotional support). Moreover, perceived support has been more commonly used allowing for between-study comparisons and shows reliable and strong associations with individual outcomes such as mental and physical health (Barrera, 1986; see also Sarason et al., 2001).

Perceived social support can, in turn, be differentiated on the basis of its source. Whereas perceived organizational support is based on employees’ conviction that their firm values their effort and cares for their wellbeing (Rhoades and Eisenberger, 2002), nonwork-based or external perceived social support is obtained from sources peripheral to the organization, including family, friends, and significant others. Over the last 20 years, studies have reported the moderating effects of employees’ perceived external support on the relationships between various occupational stressors and both work and life outcomes. For instance, scholars have found that support received from family and friends buffered employees’ overall life dissatisfaction associated with job insecurity as well as the relationship between physical and psychological stressors and anxiety (Lim, 1996; Frese, 1999). Others have found that external support mitigated the positive relationship between job insecurity and employees’ mental strain and somatic complaints (Näswall et al., 2005). Collectively, these findings point to external support’s ability to lessen the burdens of stressful workplace situations such as experiences of ostracism. One added justification for studying perceived external support rests with the logic that fulfilling one’s psychological belongingness needs from sources outside work buffers the impact of workplace ostracism. This is consistent with Baumeister and Leary’s (1995) substitution hypothesis which propounds that belongingness can be fulfilled by developing relationships with other individuals and social groups whenever they are thwarted by ostracism. In other words, the need to belong drives individuals to seek other sources from which to satisfy this need, with the implication that the damaged relationship with a given person or group could be replaced, as a source for need satisfaction, with another (see also Maner et al., 2007). Since work relationships are closer to the ostracism source and often have conflicting motives, interests, or relationships with the ostracizer, bonds forged outside work may be better suited in shoring up the threatened needs associated with workplace ostracism.

Studies dealing with how external social support networks affect people’s ability to cope with being shunned by others at work are now growing. For example, in the first field study on workplace ostracism to test Baumeister and Leary’s (1995) substitution hypothesis, Scott et al. (2014) found that ostracized employees who received high levels of support from family and friends experienced more job-related tension than those who received lower levels of the same support. This time, external social support acted not as a buffer against the detrimental effects of social exclusion but instead as an enhancer. The authors argued that social networks outside work might have increased employees’ rumination of the problems experienced at work thereby exacerbating their anxiety. While Scott et al.’s (2014) study sheds light on important psychological outcomes, what remains to be shown is how ostracized employees respond behaviorally. One behavior that we investigate is helping others at work, and we initiate this inquiry by looking at what happens to ostracizees’ degree of influence over resources following exclusion from work colleagues of varying levels of hierarchical status.

## Helping After Exclusion

Ostracizing a person involves, whether consciously or not, a change in the power relations that exist between the agent and victim. Not surprisingly, individuals who ostracize others report feeling powerful and those who are ostracized report feeling powerless (Veldhuis et al., 2014). In organizational psychology, power has been variously defined as “the ability to provide or withhold valued resources or administer punishments” (Anderson and Berdahl, 2002, p. 1362), or as “the ability to control resources, own or others’, without social interference” (Galinsky et al., 2003, p. 454). Thus, organizational scholars seek to understand the ways in which a person having power makes decisions that ultimately determine the outcomes of another person (Galinsky et al., 2003; Keltner et al., 2003). In this paper, we define power in this same tradition as the lopsided control over valued resources that some individuals possess over others (see also Fiske, 2010; Anderson and Brion, 2014). Thus, whereas status is a structural concept rooted in a social system that grants those having a high rank easier access to resources, power is a relational concept in which low-status individuals rely on high-status ones for the acquisition of rewards and the avoidance of punishments (Emerson, 1962). Although conceptually distinct, power and status are correlated such that those having a higher organizational status tend to be more powerful than those occupying a lower status (Davis and Moore, 1945). In this paper, we assume that both constructs grant the holder control over desired resources and, by extension, control over others. Since power is highly associated with one’s accumulated material and social resources (Keltner et al., 2003), being ostracized at work would, therefore, put the individual at a disadvantage when it comes to accessing organizational resources. These resources can take on several forms (e.g., informational, psychological, and financial) and drawing from them is contingent on being included in the ‘right network.’ Thus, an ostracizee would be experiencing a decrease in power, and this reduction in power could be one of the mechanisms by which ostracism is associated with prosociality. In fact, the contingent nature of an individual’s perceived and exercised power on group inclusion has received attention in the literature (Berger et al., 1972; Schwarzwald et al., 2005).

According to approach/inhibition theory of power (Keltner et al., 2003), an individual with diminished power would become sensitive to potential social threats and increasingly attentive to the actions and needs of others. Similarly, ostracized individuals are limited in their ability to access various social and material resources (Williams, 2001) and, as a result, experience a reduction in their perceived power and within-group status. One way to regain power and access to group resources is through a heightened vigilance of others and an accompanying willingness to help them (Fiske, 1993). While high-power individuals perceive others through a ‘lens of self-interest,’ ostracized individuals are more likely to see themselves through the needs of others (Keltner et al., 2003, p. 271). At work, ostracizees could enhance their influence and potential for reinclusion through the enactment of prosocial behavior, an example of which is organizational citizenship behavior. Defined as “performance that supports the social and psychological environment in which task performance takes place” (Organ, 1997, p. 95), organizational citizenship behavior can be directed either toward the organization as a whole (OCB-O) or toward individuals (OCB-I; Williams and Anderson, 1991). Examples of OCB-O include giving advance notice of one’s inability to come to work, avoiding undeserved work breaks, and adhering to informal norms that help maintain order. On the other hand, cases of OCB-I include helping other employees who have been absent or who have had intense workloads, going out of one’s way to assist new coworkers, and taking time to listen to and empathize with coworkers. Given ostracism’s interpersonal nature, we focus on OCB-I and propose that ostracizees’ loss of power and subsequent desire to regain what was lost will render them more attentive to others’ needs and thus more likely to engage in OCB-I. Through the exercise of OCB-I, ostracized employees could be seen as engaging in impression management to secure future resources and reaffirm lost power (Rioux and Penner, 2001). Some may argue that the traditional social exchange view of OCB predicts the opposite, namely that ostracizees will reduce their helping to counter violations of reciprocity (Gouldner, 1960; Blau, 1964). Research findings, however, have been mixed with some showing positive, negative, and null relationships between ostracism and prosocial behavior (for a review, see Ferris et al., 2017). A more recent perspective sees OCB as a social dilemma, or a choice between incurring short-term costs to gain long-term benefits like group reinstatement (Joireman et al., 2006; Balliet and Ferris, 2013). The fact that ostracizees respond prosocially also distinguishes ostracism from other incivility behaviors such as bullying and abuse that lead to further spirals of uncivil and retaliatory behaviors (Ferris et al., 2017). As such, we expect victims of ostracism who experience a loss of power to view OCB-I as a long-term investment strategy toward future reinclusion.

Hierarchical status gives one legitimate control, and hence power, over the distribution of resources that are both challenging to replace and prohibitive to lower-status persons. In the workplace, there often exists an imbalance of power between supervisees (or subordinates) and the supervisors (or managers) to whom they report. As such, supervisees who are socially excluded by a higher-status supervisor are particularly vulnerable to resource deprivation. As the ostracizer’s status increases vis-à-vis that of the ostracizee, access to resources becomes jeopardized thereby increasing the relative power differential between the two parties and triggering the ostracizee’s motivation to engage in OCB-I as a means to regain rank within the network. Conversely, when the differential is in the ostracizee’s favor, loss of power is minimized and the motivation for OCB-I will be lower. Research has shown that victims are less likely to help and engage in fewer free-riding behaviors after being ostracized by lower-status perpetrators compared to higher-status ones (Williams and Sommer, 1997; Hitlan et al., 2006). This logic runs counter to findings on the association between abusive supervision and citizenship behavior, which show an overall weak negative relationship (see meta-analysis by Mackey et al., 2017). Although both abusive supervision and ostracism by a higher-ranked person limit a lower-status person’s access to resources, each of these behaviors could conceivably engender a different response from victims. Whereas abuse is an intense act with clear negative intentions that should preclude victims from contemplating prosociality as a means to restore relations, ostracism’s unclear intentions and ambiguous nature could leave open such a possibility. Seeing that abuse and ostracism by one’s supervisor are qualitatively different, it would therefore be worthwhile to explore the latter’s effects on prosocial behavior directed toward others.

Victims of workplace ostracism also have the option of turning elsewhere for succor. Perceived external social support can provide replacements for lost resources of various types (e.g., social and emotional). Since exclusion is a cue by which an individual or group signals its desire to distance itself from a lower-status member, the existence of an external support network should instead validate the ostracizee’s self-worth. As such, the existence of a strong external support system that can be relied upon should reduce the negative effect of ostracism on victims. Consequently, the latter should become less attuned to the needs of coworkers and less likely to partake in OCB-I. Both boundary conditions are also believed to interact with one another to affect the relationship between an ostracizee’s exclusion and his or her engagement in OCB-I. Specifically, we argue that the ostracizer’s status can influence the extent to which the ostracizee perceives and relies on external sources of support. In the case of a high-status ostracizer (e.g., manager), the loss of a victim’s power should strengthen the salience and value of other relational resources. Being ostracized by a high-status peer is an attack on one’s social worth. One will not only try to restore what was lost by performing OCB-I, but will also become more likely to seek out belongingness needs and secure resources outside the organization. These perceived sources of external support will, in turn, affect the amount of OCB-I performed. Based on this rationale, we hypothesize the following:

H1: External social support will moderate the interaction between ostracizer status and felt ostracism on organizational citizenship behavior directed toward individuals. Specifically, an increase in ostracizee organizational citizenship behavior that is associated with increased ostracizer status will weaken when ostracizees have high external social support.

## Deviance After Exclusion

Antisocial responses, which constitute one aspect of counterproductive work behaviors, are another means by which ostracizees restore thwarted needs and diminished power in the workplace. Meta-analytic studies have shown that citizenship and counterproductive work behaviors are distinct concepts that do not represent ends of the same behavioral continuum (Dalal, 2005). In fact, research has shown that individuals can engage in citizenship behaviors and deviant acts simultaneously (Sackett et al., 2006). Therefore, it is possible that individuals can act in both pro- and antisocial ways in response to ostracism. Like citizenship behaviors, deviant acts can be separated into two empirically distinct categories (Berry et al., 2007). These include organizational deviance where actors cause harm to the entire firm (e.g., damaging company property, intentionally delaying work, and lying about hours worked) and interpersonal deviance where pain is inflicted on individuals (e.g., cursing at coworkers, gossiping about coworkers, and showing favoritism) (Robinson and Bennett, 1995). As in the case with OCBs, due to the interpersonal nature of ostracism, we focus on deviant behaviors directed toward others.

We propose that a reduction in power resulting from workplace ostracism and the subsequent loss of control over valuable resources will motivate ostracizees to attempt to regain power through deviant acts in order to restore threatened needs. While some argue that ostracism elicits negative reactions from ostracizees because it threatens one’s identity (Thau et al., 2007), others claim that antisocial responses are a conduit to reestablishing control over one’s environment (Warburton et al., 2006; Gerber and Wheeler, 2009). Individuals high in power are less inhibited when interacting with others because they are less dependent on them for sustenance and could thus be more defiant than those low in power (Keltner et al., 2003). Following this argument, ostracizees would therefore be less likely to engage in interpersonal deviance. In contrast, some scholars challenge that ostracism increases antisociality when the primary need is to restore control rather than belongingness (Williams and Wesselmann, 2011). One way of reconciling these opposing views is to turn one’s attention to human aggression and its two principal forms, reactive and proactive aggression, each of which corresponds to distinct behaviors (Poulin and Boivin, 2000; Carré et al., 2011). Individuals engage in reactive aggression whenever they experience provocation to which they retaliate with high arousal (e.g., anger) and impulsiveness. On the other hand, they may also aggress against others in the absence of provocation (i.e., proactively), with no pronounced arousal and often as a means of appropriating others’ resources (Poulin and Boivin, 2000). We argue that deviant behaviors performed against others that are induced by ostracism are reactive in nature and recent work has shown that these two forms of aggression possess different antecedents. For example, Vongas and Al Hajj (2015a, 2017) posited and found that individuals experiencing a status gain tend to aggress proactively against others, whereas those sustaining a loss tend to aggress reactively. They explained that those high in status are motivated to maintain and exercise their power through the victimization of new targets. Those having lost status, however, will hesitate to initiate aggression out of fear of further status loss. Any increase in deviance by the ostracizee could be explained as a reactive response with the goal of regaining status within the group. This explanation is consistent with approach/inhibition theory of power (Keltner et al., 2003), which holds that while the behavior of high-power individuals more closely mirrors their disposition rather than the situation, the reverse is true for low-power individuals. The context, therefore, seems to play a stronger role for lower-power individuals. As power decreases, threatening events are perceived with greater intensity (Keltner et al., 2003) and scholars have shown that ostracizees experience reduced empathy even toward other victims of ostracism (DeWall and Baumeister, 2006). As such, we expect ostracizees to be driven less by personality and more by the hostile context when contemplating workplace deviance.

The function of social support in controlling deviant behavior has also been studied extensively (Goldstein et al., 1981; Clinard and Meier, 2011). By hindering opportunities (Merton, 1938), ostracism deprives the ostracizee from resource access, increasing the likelihood that deviance will be used to restore those resources. Having strong external social support ties, however, can improve one’s access to resources and thus reduce the need for delinquency or any other form of interpersonal norm violation. An individual supported by strong external networks will still have the opportunity to satisfy belongingness needs outside of work. If deviance is believed to be a reaction prompted by threats to one’s belongingness in a work context, then having external ties should render workplace ostracism less threatening. Under such conditions, one could expect deviance to become reduced.

The higher an individual’s hierarchical status, the more he or she will be regarded as an embodiment of the organization (Levinson, 1965; Eisenberger et al., 2010) and the more this person will be expected to conform the certain behavioral norms (Hogan and Emler, 1981). As such, ostracism perpetrated by higher-status individuals will be especially hurtful because authority figures are expected to be helpful and to represent the organization positively. As a consequence of this treatment and the reduced access to organizational resources, we anticipate increased deviance from ostracizees. Unlike in the case of OCB-I, we expect that being ostracized by a supervisor will lead to an increase in deviant behaviors similar to the increase associated with supervisory abuse (Mackey et al., 2017). The reason is that ostracism-induced OCB-I and deviance each serves a different purpose. While the former is an attempt to polish one’s image and demonstrate that he or she is a valuable member worth group reinclusion, the latter is a reactive form of aggression whose goal is to re-establish lost power. Again, comparing how abuse and ostracism differ with respect to deviant behaviors remains an important and unanswered question. While both should lead to deviance, which of the two will result in a more aggravated response is difficult to predict. As abuse is more intense and well defined than ostracism, it nevertheless acknowledges the existence and importance of the victim, hence posing less of a threat to his or her need to belong.

Perceived low status has also been associated with reduced discretionary behavior, which increases the probability of retaliation (Aquino et al., 2001; Aquino and Douglas, 2003). Since reactive deviance following ostracism can be seen as an attempt to regain control, ostracizees would engage in more interpersonal deviance after being ostracized by a higher-status authority due to the greater loss of power and resource accessibility. The importance of jointly considering the ostracizer’s status and the ostracizee’s perceived external support together should, again, not be overlooked. The adverse behavioral effect of resource and power losses due to rejection by a high-status individual could be buffered if the victim has access to external social support. For example, studies have shown that external support can increase one’s self-esteem (Brown et al., 1986) that could, in turn, reduce one’s proneness to being ostracized (Leary and Downs, 1995) as well as one’s overall sensitivity to poor interpersonal treatment (Avey et al., 2011). Given the above, we hypothesize the following:

H2: External social support will moderate the interaction between ostracizer status and felt ostracism on interpersonal deviance. Specifically, an increase in ostracizee interpersonal deviance that is associated with increased ostracizer status will weaken when ostracizees have high external social support.

## Ostracism and the Intention to Quit

Turnover intention, defined as “the conscious and deliberate willfulness to leave the organization” (Tett and Meyer, 1993, p. 262), has a long history in the workplace literature. This interest stems partly from the fact that turnover intention is widely hailed as a strong predictor of actual turnover (Steel and Ovalle, 1984). As such, knowing what factors influence the intention to quit is critical to understanding turnover and its repercussions. In the workplace, feelings of social disconnection and ostracism may increase this desire as one’s sense of obligation toward coworkers and satisfaction with, and commitment to, the organization begin to erode (Harkins and Petty, 1982; Hitlan et al., 2006). Turnover intention constitutes part of a defensive withdrawal mechanism that protects the individual from the psychological pain associated with being ostracized (Ferris et al., 2008). Thoughts of leaving the organization could also appease an individual’s concern that coveted resources lost via ostracism could be secured elsewhere. As Hobfoll (1989) noted, the loss of coveted resources induces stress. Since resource replacement is the most direct way to offset this loss, the intention to quit can be an initial stage in which ostracizees contemplate seeking new employment where lost status and resources can be restored (Maner et al., 2007).

Consistent with this rationale is research showing a positive relationship between workplace ostracism and turnover intention as well as job-seeking behavior (Ferris et al., 2008). More recently, studies have also demonstrated that perceptions of workplace ostracism are directly related to both turnover intention and actual turnover (Renn et al., 2013; O’Reilly et al., 2015). This body of work suggests that ostracism victims will express a greater desire to abandon their position compared to included employees (O’Reilly et al., 2015). It stands to reason, however, that ostracizer status and ostracizee external support may influence turnover intention. According to our reasoning, being ostracized by someone of higher status (e.g., a supervisor) compared to one of lower status (e.g., a subordinate) leads to a greater loss of resources making the prospect of leaving more attractive. A high-status ostracizer also has the potential to influence others into excluding the target. As one’s hierarchical status increases, others will come to view this individual as an organization’s ambassador and his or her behaviors will be translated into attitudes toward the firm (Levinson, 1965; Eisenberger et al., 2010). Being ostracized by a higher-status individual will also reduce one’s ability to perform, consequently lowering the expected return from the job and producing a more negative attitude toward the organization than if the ostracizer was of equal or lower status. While the specific mechanism is speculative at the moment, such a cascade of events could increase one’s intention to quit.

Experimental work has shown that ostracized individuals are motivated to form bonds with external sources of affiliation (Maner et al., 2007). Hence, external social support is expected to be another important factor affecting the ostracizee’s turnover intention. To better understand the association between social support and voluntary turnover, however, requires looking beyond traditional attitude models. Mitchell et al. (2001, p. 1104) define job embeddedness as a “broad constellation of influences on employee retention” comprised of three dimensions. First are ‘links’ which represent the number and strength of formal and informal connections between a person and others at work. Second is ‘fit’ which takes into account one’s level of comfort with a given job and how this job fits his or her life space. And third is ‘sacrifice’ which represents what a person will forfeit if he or she decides to leave the job (Mitchell et al., 2001; Lee et al., 2004). Being less embedded renders an employee more sensitive to shock and dissatisfaction and thus more willing to contemplate quitting (Holtom and Inderrieden, 2006). Ostracism would appear to have negative effects on both the links and fit dimensions. The extent to which it would have a negative effect on sacrifice, however, depends on an ostracizee’s level of external social support. This is one way in which perceived external support is related to job embeddedness. Strong social ties will make the option of leaving less threatening and reduce current job dependence due to a built reservoir of obtainable resources. As such, both experienced workplace ostracism and a strong external social support should positively influence one’s intention to voluntarily quit.

Again, we believe it is critical to consider the effects of both the ostracizer’s hierarchical status and the ostracizee’s external social support in tandem when looking at the connection between felt ostracism and turnover intention. Being ostracized by a high-status individual might not be able to sufficiently elicit the intention to abandon one’s job if the potential loss anticipated by quitting is too high. Examples abound in which employees are willing to tolerate negative interpersonal treatment because of the high level of sacrifice associated with quitting, such as limited advancement opportunities and increased family demands (Maertz and Kmitta, 2012). While relying on one’s external social support network could widen the possibilities of other job prospects, such support again might fail to induce one’s intention to quit when ostracizer status is low. In this case, although forfeited resources would be insignificant because low-status ostracizers contribute few, if any, of them in the first place, job embeddedness could still be significant due to links with influential individuals. Moreover, perceived fit with the organization may still be strong because low-status ostracizers will not adversely affect one’s job comfort thus reducing the intention to voluntarily quit. Therefore, we hypothesize:

H3: External social support will moderate the interaction between ostracizer status and felt ostracism on turnover intention. Specifically, an increase in ostracizee turnover intention that is associated with increased ostracizer status will strengthen when ostracizees have high external social support.

## Overview of Studies

Recent work has demonstrated that vignettes are an effective method of examining workplace ostracism (Balliet and Ferris, 2013). Therefore, we employed an experimental vignette methodology (Aguinis and Bradley, 2014) and designed Study 1 as a between-subjects investigation in which participants immersed themselves in a workplace scenario that manipulated their level of ostracism, their ostracizer’s hierarchical status, and their level of external social support. Participants were then asked to report their willingness to engage in organizational citizenship behaviors directed toward individuals (OCB-I), interpersonal deviance (I-Dev), and turnover intention. In Study 2, we attempted to generalize findings from Study 1 through a field study where working adults indicated their level of workplace ostracism and external social support, and specified their ostracizer’s status while reporting the same outcome variables 1 week later.

## Study 1 Method

### Participants and Procedure

Three hundred and twenty eight adults (134 female or 40.85%) with an average age of 33.61 years (*SD* = 11.75) were recruited via Amazon Mechanical Turk (MTurk) to take part in a study about workplace experiences. The sample came predominantly from the sales and service sectors (23%), followed by education, government, and social services (22%). A verification of IP address locations ensured that all participants were based in the United States. We underscored that participation was voluntary and all information provided would remain anonymous. Each participant was paid US $1.50 through individual MTurk codes to ensure anonymity. Because the manipulation involved a work situation, we included only participants who were employed within the last 12 months resulting in an average organizational tenure of 3.12 years for the final sample (*SD* = 1.07). After providing informed consent, participants completed a short demographic questionnaire and read a vignette describing an ostracism episode at work. Each vignette described how the participant worked for a major advertising firm for the past 2 years, specializing in the development of social media campaigns for caffeinated products. The story then introduced a work colleague and mentioned his role within the firm and his subsequent treatment of the respondent. Finally, the respondent’s relationships with family, friends, and significant others were specified after which participants provided self-reports of our three focal outcome variables.

### Vignette Scenarios

Twelve (12) vignettes were developed using a 2 × 2 × 3 factorial between-subjects design in which we manipulated levels of workplace exclusion (ostracism versus inclusion), external social support (low versus high), and relative ostracizer status (lower versus equal versus higher status). Through the randomization feature of the Qualtrics^TM^ survey suite, participants were randomly assigned to 1 of the 12 work scenarios involving an interaction with a protagonist named Edward. All participants were described as having the same professional advertising position, whereas Edward’s role varied to reflect different degrees of relative hierarchical status. In the ostracism manipulation, they read that Edward ‘never answers your phone calls or emails,’ ‘gives you the cold shoulder when you meet,’ and ‘has never invited you to any of the after-work social events that he organizes.’ By contrast, in the inclusion manipulation, they were told that Edward ‘promptly responds your phone calls or emails,’ ‘makes sure to consistently keep you in the loop,’ and ensures that ‘you are always invited’ to social gatherings.

Second, Edward’s organizational role was adapted to include one of three status levels: advertising copywriter (lower status), advertising professional (equal status), and advertising manager (higher status). This information on each party’s relative status was incorporated in the vignettes in various ways, such as ‘Edward, an advertising copywriter whom you directly supervise...’ or ‘As your subordinate, Edward consults you….’

Finally, information about the participant’s level of external social support was specified as being either high or low. This manipulation was based on the Multidimensional Scale of Perceived Social Support (Zimet et al., 1988) which assesses support derived from family, friends, and significant others. In the low-support condition, participants were informed that ‘You have been estranged from your family for the past few years,’ ‘You have no current dating prospects,’ and ‘Overall, you feel that you are lacking a strong support system outside of work and you often feel lonely.’ In contrast, participants in the high-support condition were told that ‘You are very close with your parents and have a very good relationship with them,’ ‘You have recently started dating and things have been going really well,’ and ‘Overall, you have a very positive social life outside of work and you feel loved and supported by your friends and family.’ After reading the vignette, participants were asked to rate their inclination to voluntarily assist coworkers in tasks (OCB-I), as well as their likelihood of acting out various adverse behaviors toward them (I-Dev), and their intentions to quit.

### Measures

#### Experienced Ostracism

The extent to which participants felt excluded was measured using a single item: “How excluded did you feel by your work colleague?” This item was measured on a seven-point Likert scale from 1 (*not at all*) to 7 (*extremely*). Recent evidence suggests that participant responses from scales comprised of singular items can be both valid and reliable (Postmes et al., 2013), particularly in cases where the item is designed to assess a homogeneous and clearly defined construct. Therefore, for both conceptual and pragmatic reasons, we opted to use this measure.

#### Ostracizer Status

The hierarchical status of the focal employee in each vignette was verified by asking participants to correctly identify their rank relative to Edward’s using the following three choices: of lower status, of equal status, or of higher status.

#### Perceived External Social Support

The level of external social support experienced by participants was measured using the following item: “How supported do you feel outside of work?” This single item was measured using a seven-point Likert scale ranging from 1 (*not at all*) to 7 (*extremely*).

#### Organizational Citizenship Behaviors Directed toward Individuals

The willingness with which employees preferred to behave positively toward members of their workgroup was measured using a version of Lee and Allen’s (2002) individual-directed Organizational Citizenship Behavior Scale. This scale was modified to fit the vignette stories. Using a seven-point Likert scale ranging from 1 (*never)* to 7 (*always*), respondents were asked to indicate the extent to which they would participate in these behaviors assuming they took on the role of the vignette’s focal employee. A sample item is: “I would help members of my team who have been absent” (Cronbach’s α = 0.90).

#### Interpersonal Deviance

To measure participants’ willingness to partake in deviant behaviors directed at their coworkers, we used a modified version of Bennett and Robinson’s (2000) seven-item Interpersonal Deviance Scale ranging from 1 (*never*) to 7 (*very often*). A sample item is: “I would act rudely toward members of my workgroup” (Cronbach’s α = 0.96).

#### Turnover Intention

The extent to which participants wanted to leave their position was measured using Colarelli’s (1984) Intent to Quit Scale, a three-item scale ranging from 1 (*strongly disagree*) to 6 (*strongly agree*). A sample item is: “If I have my own way, I will not be working for this organization 1 year from now” (Cronbach’s α = 0.94).

### Study 1 Analysis and Results

#### Attention and Manipulation Checks

To ensure that respondents read their assigned vignette thoroughly, we disabled the forward button for 60 s and asked them to answer two questions on the story’s content following the reading. One question asked them to identify their hypothetical firm’s name from three similar-sounding names and the other question asked for their position within the organization. Results indicated that 95% of participants accurately responded to the question pertaining to the name of the focal organization, while 94% correctly indicated the name of their position. A total of 33 individuals committed errors on either one of these two checks and were removed from further analyses, leaving a final sample of 295 participants.

To measure the effectiveness of each condition, we performed three one-way between-subjects ANOVAs. For the manipulation of the ostracizer’s status relative to the respondent’s, there was a significant effect of the condition [*F*(2,293) = 218.77, *p* < 0.001]. A Tukey HSD test indicated that participants in the lower-status ostracizer (*M* = 1.30; *SD* = 0.63), similar-status ostracizer (*M* = 2.10; *SD* = 0.38), and higher-status ostracizer conditions (*M* = 2.77; *SD* = 0.53) all differed significantly from one another (*p <* 0.05). Included participants reported feeling more accepted (*M* = 6.36; *SD* = 0.91) following the vignette’s completion than ostracized participants [*M* = 3.42; *SD* = 2.15; *F*(1,294) = 96.60, *p* < 0.001]. Furthermore, participants in the high external social support condition (*M* = 6.63; *SD* = 1.75) reported feeling more supported than those in the low-support condition [*M* = 2.82; *SD* = 2.08; *F*(1,294) = 509.69, *p* < 0.001]. These results indicated that individuals were able to accurately discern the differences between each of the three manipulations.

#### Hypothesis Testing

**Table 1** presents the means for each of the dependent variables in each of the 12 conditions. Correlations between the variables of Study 1 are shown in **Table 2**.

**Table 1:** Study 1: Means for the 12 vignette scenario conditions.

Condition			OCB-I		I-Dev		Turnover Intentions	
		*N*	Mean	*SD*	Mean	*SD*	Mean	*SD*
**Inclusion conditions**					
Lower status	Low external support	27	5.27	0.86	1.52	0.76	1.84	1.10
	High external support	24	5.80	0.86	1.61	0.75	2.38	1.26
Similar status	Low external support	25	5.42	1.05	1.65	0.82	2.27	1.33
	High external support	26	5.75	1.06	1.54	0.62	2.96	1.73
Higher status	Low external support	24	5.59	0.74	1.39	0.38	2.60	1.53
	High external support	22	6.01	1.01	1.63	0.74	2.14	1.34
**Ostracism conditions**					
Lower status	Low external support	27	4.67	1.17	2.02	0.72	2.98	1.26
	High external support	28	4.77	1.40	1.83	1.00	2.98	1.40
Similar status	Low external support	23	4.59	1.42	2.15	1.03	3.74	1.31
	High external support	22	4.42	1.90	1.86	0.64	3.13	1.27
Higher status	Low external support	23	3.89	1.44	2.71	1.19	3.94	1.64
	High external support	24	4.75	1.19	1.80	0.83	3.41	1.38

*N = 295. OCB-I, organizational citizenship behavior-individual; I-Dev, interpersonal deviance.*

**Table 2:** Study 1: Descriptive statistics, zero-order correlations, and alphas.

	Mean	*SD*	1	2	3	4	5	6	7	8	9
(1) Age	33.61	11.75	–								
(2) Gender	0.41	0.49	0.17^∗^	–							
(3) Tenure	3.12	1.07	0.23^∗∗^	–0.08	–						
(4) Ostracism	–	–	–0.02	–0.01	0.05	–					
(5) External social support	–	–	–0.02	0.02	–0.07	–0.01	–				
(6) Status	–	–	–0.09	0.03	–0.02	0.02	0.04	–			
(7) OCB-I	5.07	1.31	0.17^∗^	0.13^∗^	0.01	–0.36^∗∗^	0.01	–0.06	**0.90**		
(8) Turnover intentions	2.87	1.49	–0.19^∗∗^	–0.02	–0.07	0.33^∗∗^	–0.02	0.14^∗^	–0.36^∗∗^	**0.94**	
(9) I-Dev	1.56	0.87	–0.20^∗∗^	–0.15^∗^	–0.00	0.23^∗∗^	–0.11^∗^	0.06	–0.49^∗∗^	0.45^∗∗^	**0.96**

*N = 295. Status, hierarchical status of ostracizer; OCB-I, organizational citizenship behavior-individual; I-Dev, interpersonal deviance.*

*^∗^*p <* 0.05, ^∗∗^*p <* 0.01. Cronbach’s alphas in bold.*

**FIGURE 1:**
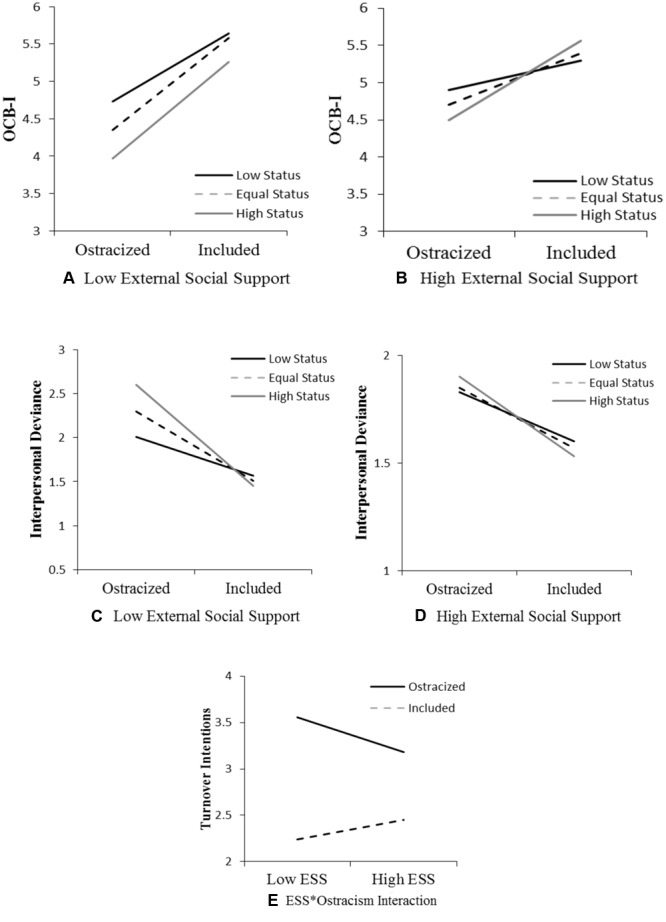
Study 1: Moderation effects of ostracizer status and external social support. OCB-I, organizational citizenship behavior-individual; ESS, external social support.

To test our hypotheses, we used Hayes’s (2013) conditional process model (version 2.12). This model makes simultaneous calculations of all paths possible and effectively handles the non-normality of interaction terms through the use of bootstrapping and repeated sampling with replacement. We tested the three-way interaction models (Model 3 in Hayes, 2013) for each of the hypothesized outcomes (OCB-I, I-Dev, and turnover intention) such that the relationships between ostracism and the outcome variables would be moderated by both the ostracizee’s external social support and the ostracizer’s hierarchical status. Results are displayed in **Table 3** and interaction plots are illustrated in **Figure 1** at low and high levels of external social support.

**Table 3:** Study 1: Regression results for three-way interaction outcomes.

Variable	OCB-I				I-Dev				Turnover intentions			
	*B*	*SE*	*t*	*p*	*B*	*SE*	*t*	*p*	*B*	*SE*	*t*	*p*
Constant	5.66	0.21	26.02	0.00	1.57	0.14	10.95	0.00	1.86	0.25	7.54	0.00
Status	–0.07	0.17	–0.45	0.65	–0.07	0.10	–0.63	0.53	0.38	0.19	2.01	0.05
Ostracism	–0.85	0.31	–2.73	0.01	0.36	0.18	2.01	0.05	1.22	0.35	3.46	0.01
Status × Ostracism	0.38	0.21	2.00	0.05	0.42	0.16	2.69	0.01	0.10	0.27	0.38	0.71
Support	–0.33	0.30	–0.38	–0.71	–0.28	0.24	–0.40	0.67	0.72	0.35	2.09	0.04
Ostracism × Support	0.24	0.43	0.54	0.58	–0.11	0.28	–0.38	0.71	–0.84	0.49	–2.04	0.05
Status × Support	0.87	0.24	0.73	0.71	0.07	0.16	0.46	0.65	–0.46	0.27	–1.73	0.10
Ostracism × Status × Support	–0.65	0.31	–2.03	0.05	0.43	0.22	2.04	0.05	0.20	0.38	0.53	0.59

*N = 295. Status, hierarchical status of ostracizer; Support, perceived external support.*

Although the test for Hypothesis 1 found a significant three-way interaction of ostracism, external social support, and ostracizer hierarchical status on OCB-I (*B* = -0.65, *t* = -2.03, *p* < 0.05), further inspection of the means and graphs in **Table 1** and **Figure 1**, respectively, shows that this hypothesis was not supported. In all groups, ostracized individuals engaged in fewer OCB-I than included ones, and in all groups except one – the similar-status ostracized group – individuals having low external social support engaged in fewer OCB-I than those having high external support. In addition, with increasing levels of ostracizer status, individuals with both low and high external social support experienced a drop in OCB-I (**Figures 1A,B**). Consistent with our prediction, however, we found support for Hypothesis 2 as evidenced by a significant three-way interaction of ostracism, external social support, and ostracizer’s status on I-Dev (*B* = 0.43, *t* = -2.04, *p* < 0.05). Finally, we found no support for Hypothesis 3 as indicated by a non-significant three-way interaction between ostracism, external social support, and ostracizer’s status on turnover intention (*B* = 0.20, *t* = 0.53, *p* = 0.59). Supplementary analysis, however, revealed a significant two-way interaction between ostracism and social support for turnover intention (*B* = -0.84, *t* = 2.04, *p* < 0.05).

#### Study 1 Brief Discussion

Findings of Study 1 provide strong support for the prediction that ostracizer hierarchical status and ostracizee external social support are important determinants in the effect of workplace ostracism on an ostracizee’s subsequent behaviors and intentions. Although the direction of the relationship predicted in Hypothesis 1 was not supported, a significant three-way interaction suggests that the ostracizee’s external support and the ostracizer’s status are influential when it comes to victims’ interpersonal helping behavior. Individuals were less willing to participate in OCB-I when ostracized, and this negative relationship became stronger with decreases in external social support and increases in ostracizer status. Tests of Hypothesis 2 again showed that the two boundary conditions influenced interpersonal deviance following exclusion in the predicted direction. Individuals with the lowest perceived support who had been ostracized by someone of higher status indicated the strongest propensity for deviance toward others (see **Table 1** and **Figures 1B,D**). We found no support for Hypothesis 3, which posited that external social support and status would simultaneously affect the relationship between ostracism and turnover intention. However, a two-way interaction unveiled that external support weakened the relationship between ostracism and intentions to quit such that ostracized individuals with more perceived support reported less intent to leave the organization (see **Table 1** and **Figure 1E**). To test whether our Study 1 findings would replicate in actual experiences of workplace ostracism, we designed a field study (Study 2) involving employed adults.

## Study 2 Method

### Participants and Procedure

Using a snowball sampling procedure, we had graduate students from a Canadian university recruit 301 full-time adult workers from their social network (97 female or 32.23%) to partake in a study on workplace experiences, in exchange for course credit. Respondents had an average age of 30.24 years (*SD* = 9.27) and an average organizational tenure of 4.11 years (*SD* = 4.61). All respondents were informed that participation was voluntary. After providing informed consent, they completed two questionnaires 1 week apart. From the 301 recruits, 232 (78 females or 33.62%) completed both questionnaires for a response rate of 77.07%. No significant demographic differences were found between respondents and non-respondents, suggesting no systematic differences between the two groups. To minimize common method bias (Podsakoff et al., 2003), we first informed participants that responses would be kept confidential to reduce potential social desirability. Second, we separated the data collection of independent and dependent variables to minimize self-report bias. As such, measures of workplace ostracism, status of the ostracism source, and external social support were collected in the first survey and the three dependent variables of OCB-I, I-Dev, and turnover intention were collected in a separate survey distributed 1 week later.

### Measures

#### Workplace Ostracism

Perceived ostracism at work was measured using the 10-item Workplace Ostracism Scale (Ferris et al., 2008). Participants were asked to report on various kinds of workplace ostracism that they had faced over a 6-month period. A sample item is: “Others refused to talk to you at work.” Each item was measured on a seven-point Likert scale ranging from 1 (*never)* to 7 (*always*) (Cronbach’s α = 0.95).

#### Perceived External Social Support

Social support outside work was assessed using the 12-item Multidimensional Scale of Perceived Social Support (Zimet et al., 1988) which assesses support from family, friends, and significant others using a seven-point Likert scale from 1 (*strongly disagree*) to 7 (*strongly agree*). Two sample items are: “My family really tries to help me” and “I can talk about my problems with my friends” (Cronbach’s α = 0.92).

#### Ostracizer Status

The status of the ostracizer was measured using a single item asking all those who had experienced any form of workplace ostracism whether the initiator was of lower, equal, or higher relative hierarchical status.

#### Organizational Citizenship Behaviors Directed toward Individuals

Similar to Study 1, OCB-I was measured using Lee and Allen’s (2002) eight-item Organizational Citizenship Behavior Scale that uses a seven-point scale ranging from 1 (*never)* to 7 (*always*). Other sample items include: “I show genuine concern and courtesy toward coworkers, even under the most trying business or personal situations” and “I willingly give my time to help others who have work-related problems” (Cronbach’s α = 0.84).

#### Interpersonal Deviance

As in Study 1, Bennett and Robinson’s (2000) seven-item Interpersonal Deviance Scale was used to measure deviant behavior, ranging from 1 (*never)* to 7 (*daily*). Two other sample items are: “I have publicly embarrassed someone at work” and “I have made fun of someone at work” (Cronbach’s α = 0.86).

#### Turnover Intention

Again, similar to Study 1, we used Colarelli’s (1984) Intent to Quit Scale that ranges from 1 (*strongly disagree*) to 6 (*strongly agree*). Another sample item is: “I am planning to search for a new job during the next 12 months” (Cronbach’s α = 0.92).

#### Controls

We controlled for respondent age and gender because existing research has linked demographic characteristics to employee manifestations of harmful behaviors (Aquino and Douglas, 2003). We also controlled for tenure because meta-analytic studies have established its association with turnover (Griffeth et al., 2000). Gender was dummy-coded with male coded as “1” and female coded as “0”, while age and tenure were reported in number of years.

### Study 2 Analysis and Results

#### Common Method Bias Check

Harman’s one-factor test and confirmatory factor analysis (CFA) were used to explore whether our results were susceptible to common method bias. Accordingly, common method variance might be present if an exploratory factor analysis (EFA) produces one factor that accounts for more than half of the covariance among the variables. In our case, four factors each with an eigenvalue larger than 1.0 emerged from the unrotated EFA, accounting for 67.39% of the variance. The largest of the four retained factors accounted for less than half the overall variance (21.23%). We then performed a CFA with all variables loading on one factor. If such a model showed acceptable fit, one might suspect possible common method bias. The CFA showed that a single-factor model did not fit the data well [χ^2^(119) = 1255.10, *p* = 0.00, GFI = 0.57; CFI = 0.44; TLI = 0.35; RMSEA = 0.20]. Although these attempts do not completely rule out the possibility of common method bias, they nevertheless bolster data integrity.

#### Hypothesis Testing

Descriptive statistics, correlations, and internal consistencies of the variables in Study 2 are displayed in **Table 4**.

**Table 4:** Study 2: Descriptive statistics, zero-order correlations, and alphas.

	Mean	*SD*	1	2	3	4	5	6	7	8	9
(1) Age	30.79	9.86	**–**								
(2) Gender	1.34	0.47	0.01	**–**							
(3) Tenure	4.31	4.94	0.51^∗∗^	–0.05	**–**						
(4) Ostracism	1.68	0.92	–0.01	0.07	–0.01	**0.95**					
(5) External social support	5.19	1.19	0.09	–0.05	0.12	–0.42^∗∗^	**0.93**				
(6) Status	1.88	0.81	–0.09	0.01	–0.09	0.03	–0.03	**–**			
(7) OCB-I	5.41	0.85	0.08	0.09	0.11	–0.35^∗∗^	0.25^∗∗^	0.14^∗^	**0.84**		
(8) Turnover intentions	3.12	1.51	–0.23^∗∗^	–0.05	–0.21^∗∗^	0.30^∗∗^	–0.11	–0.05	–0.25^∗∗^	**0.92**	
(9) I-Dev	1.74	0.95	–0.11	–0.16^∗^	–0.09	0.36^∗∗^	–0.19^∗^	–0.16^∗^	–0.29^∗∗^	0.20^∗∗^	**0.86**

*N = 232. Status, hierarchical status of ostracizer; OCB-I, organizational citizenship behavior-individual; I-Dev, interpersonal deviance.*

*^∗^*p <* 0.05, ^∗∗^*p <* 0.01. Cronbach’s alphas in bold.*

**FIGURE 2:**
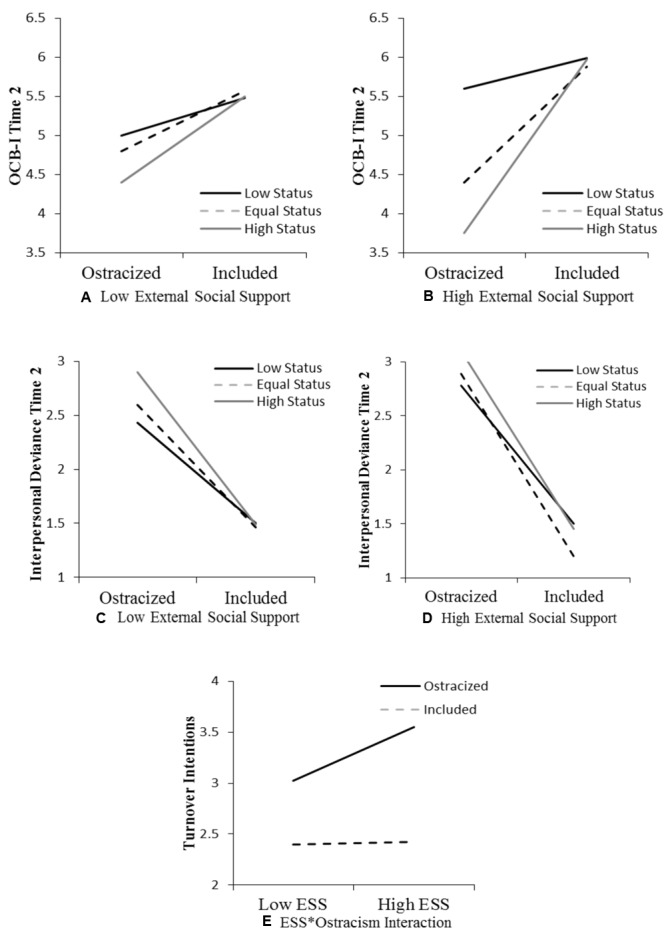
Study 2: Moderation effects of ostracizer status and external social support. OCB-I, organizational citizenship behavior-individual; ESS, external social support.

Similar to Study 1, we used Hayes’s (2013) conditional process model to test hypotheses (version 2.12; Model 3). Results are shown in **Table 5**. Consistent with Study 1, we found a significant three-way interaction between ostracism, the ostracizee’s external social support, and the ostracizer’s hierarchical status (*B* = -1.20, *t* = -2.70, *p* < 0.01), although the directions of the relationships again did not confirm predictions. Ostracized individuals reported fewer OCB-I than included individuals and their helping behaviors decreased with increases in ostracizer status (see **Figures 2A,B**). As such, Hypothesis 1 was not supported. We were also able to replicate Study 1’s findings for Hypothesis 2 as shown by a significant three-way interaction between ostracism, ostracizee external social support, and ostracizer status for I-Dev (*B* = 1.29, *t* = 3.63, *p* < 0.00). Finally, Hypothesis 3 was not supported. The three-way interaction of ostracism, ostracizee external social support, and ostracizer status was non-significant for turnover intention (*B* = -0.95, *t* = -1.32, *p* = 0.19). However, *post hoc* analyses again showed a significant two-way interaction of ostracism and ostracizee external support for turnover intention (*B* = 2.26, *t* = 2.01, *p* < 0.05). Plots for the interactions are displayed in **Figure 2**.

**Table 5:** Study 2: Regression results for three-way interaction outcomes.

Variable	OCB-I				I-Dev				Turnover intentions			
	*B*	*SE*	*t*	*p*	*B*	*SE*	*t*	*p*	*B*	*SE*	*t*	*p*
Constant	3.51	0.69	5.06	0.00	1.35	0.55	2.45	0.02	3.06	1.15	2.67	0.01
Status	1.00	0.32	3.15	0.02	–0.20	0.25	–0.81	0.42	0.12	0.46	0.27	0.79
Ostracism	–0.78	0.28	2.73	0.01	0.25	0.18	1.33	0.18	–0.38	0.52	–0.72	0.47
Status × Ostracism	–0.49	0.13	–3.87	0.00	0.21	0.10	2.12	0.04	0.18	2.08	0.87	0.38
Support	–0.85	1.56	–0.54	0.59	2.56	1.24	2.01	0.04	–5.08	2.49	–2.04	0.04
Ostracism × Support	2.03	1.01	2.00	0.05	–2.41	0.81	–2.98	0.01	2.26	1.23	2.01	0.04
Status × Support	0.86	0.70	1.23	0.22	–1.46	0.55	–2.63	0.01	2.21	1.62	1.36	0.18
Ostracism × Status × Support	–1.20	0.45	–2.70	0.01	1.29	0.35	3.63	0.00	–0.95	0.72	–1.32	0.19

*N = 232. Status, hierarchical status of ostracizer; Support, perceived external support.*

### Study 2 Brief Discussion

In Study 1, respondents assumed the role of an employee who was either ostracized or included and were asked to report their willingness to help or harm their peers, as well as describe their intentions to quit. In Study 2, individuals reported similar responses with regard to experienced ostracism at work. This study was designed to increase both the ecological validity and the generalizability of Study 1’s findings. Results resembled those of Study 1 adding credibility to the idea that the hierarchical status of the ostracizer and the victims’ external social support are key boundary conditions that should be considered when analyzing victim outcomes of social exclusion. Tests of Hypothesis 1 showed a significant three-way interaction between ostracism, the hierarchical status of the ostracizer, and the perceived external social support of the ostracizee. However, as in Study 1, this hypothesis was unsupported as the relationships were significant albeit not in the predicted direction. Ostracized employees engaged in fewer OCB-I and this relationship was stronger when they were excluded by a higher-status member and had a less supportive external social network (see **Figures 2A,B**). Hypothesis 2 was confirmed, demonstrating that deviant behaviors following ostracism intensify with increases in offender status and decreases in external support (see **Figures 2C,D**). Tests for Hypothesis 3 produced an interesting finding. Although we failed to replicate a significant three-way interaction as in Study 1, we nevertheless found a significant two-way interaction between ostracism and ostracizee external social support on turnover intention. This time, the direction was aligned with our rationale and opposite to that of the interaction in Study 1. Individuals having a strong external support network reported a stronger predilection for quitting the organization in which they were the target of ostracism than those having a weak external support system (see **Figure 2E**).

## General Discussion

### Theoretical Contributions and Practical Implications

Our aim from two studies, one using an experimental vignette and the other a field study, was to better understand how victimized workers react to ostracism in light of their external social support and the hierarchical status of the person who is ostracizing them. Specifically, we chose to look at interpersonal forms of citizenship and deviant behaviors, in addition to turnover intentions corresponding to tend-and-befriend, fight, and flight strategies, respectively. Therefore, these dependent variables were chosen because they each represent a category of outcomes traditionally associated with ostracism experiences (Williams, 2007). Scholarly research on workplace ostracism has relied on two principal theoretical frameworks. The first is the need to belong theory (Baumeister and Leary, 1995) which proposes that excluded individuals either attempt to restore relations within the ostracizing source or gravitate to other external sources for support and need fulfillment. The second is interdependence theory (Rusbult and Van Lange, 2003; Ferris et al., 2017) which explains ostracism by considering the extent that individuals and groups are mutually connected to one another. Since interdependence implies that individuals must coordinate one another’s actions to accomplish a goal, power relationships are therefore both an important and overlooked lens through which we believe social exclusion at work should be investigated.

To our knowledge, the ostracism literature is currently void with respect to applications of theories of power. This is not the case for the related literature on incivility (see Lim and Lee, 2011). We contend that the experiences and outcomes of ostracism, like incivility (e.g., condescension) will differ according to the status of the perpetrator. As such, we drew on the approach/inhibition theory of power (Keltner et al., 2003) to argue that ostracizees would experience a reduction in their power and access to valuable resources if they suffered social exclusion from a higher-status organizational member. At the same time, however, victims could regain some resource control through external outlets (e.g., family and friends). Hence, we expected that having a strong external social support base would curtail their engagement in OCB-I, whereas being ostracized by a high-status peer from within the organization would increase such behaviors. Contrary to what we predicted, however, both studies found that ostracized individuals experienced a decrease in OCB-I, and this negative relationship was exacerbated for victims who were shunned by a high-status ostracizer and who simultaneously perceived low levels of external social support. One explanation for these consistent results comes from a reconsideration of the nature of power into its two conceptual forms, personalized and socialized power (McClelland, 1975). While personalized power stems from the drive to influence others for self-serving or self-aggrandizing purposes, socialized power is achieved mostly through prosocial acts directed toward others’ benefit. In the current context, OCB-I are unsolicited prosocial helping behaviors. Any decrease in power following ostracism, including socialized power, can lead to an accompanying decrease in OCB-I. Ostracism generally leads to resource deprivation and, the fewer resources an individual possesses, the fewer resources he or she will have to invest in the form of OCB-I.

Conditions that exaggerate the disparity of power between interacting parties, such as the level of ostracizer status and the absence of a dependable external support network will lead to a sharper decline in OCB-I. Any reduction in power that serves as a modulator for reactive aggression (Vongas and Al Hajj, 2015a, 2017) will prompt more deviance and less prosociality following exclusion. Therefore, it appears that ostracizees withdraw their help not only from those individuals who excluded them (cf. Balliet and Ferris, 2013), but also from innocent members of their team. Reduced prosociality and increased deviance might further poison relationships between employees leading to even more ostracism and overt forms of abuse such as verbal or physical aggression. Given that citizenship behaviors comprise informal, voluntary, and spontaneous acts of helping, their reduction would render an organization less effective (for a review, see Podsakoff and MacKenzie, 1997).

When it comes to victims’ deviant behaviors toward others following ostracism, we predicted that a high-status ostracizer and a low level of external social support would jointly enhance the enactment of deviance. Such a finding lends credence to studies showing the positive relationship between abusive supervision and interpersonal deviance (Mitchell and Ambrose, 2007; Lian et al., 2012) and the buffering effect of external social support on negative outcomes emanating from work stressors (Lim and Lee, 2011). Finally, it also suggests that organizations might do well to rethink how their employees connect with external stakeholders. For example, providing opportunities for employees to become involved in their communities or augmenting family assistance programs are two simple ways. For multinational firms, it may be beneficial to help employees transfer to locations that reunite them with disconnected loved ones. Because external support played an important role in reducing deviant behaviors and increasing citizenship behaviors following ostracism across both studies, ensuring that employees have a strong social base outside work will benefit them and their organizations alike. It is likely, however, that facilitating external support will come at the cost of risking higher turnover intention if ostracism is not controlled. Organizations may also wish to reconsider how status differences are communicated in day-to-day affairs and promote, where possible, shared leadership. According to Katz and Kahn (1978), team members become more resourceful, share more information, and exhibit higher commitment with their team whenever they act as leaders. This configuration enables resources to be more equally distributed from one central agent, often a higher-status member of the team (e.g., a manager), to others in a way that mitigates power differences between members. A recent meta-analysis has shown that shared leadership is, in fact, positively associated with team performance (D’Innocenzo et al., 2016).

Despite its influence in determining how an ostracism experience translates to ostracizees’ citizenship and deviance behaviors, the ostracizer’s hierarchical status played no role in their turnover intention. We argued that, as the status of the ostracizer increases, the ostracizee’s resources would be increasingly reduced in both quantity and quality thus inciting the victim to ponder quitting in order to secure resources elsewhere. However, other factors could have dampened this effect. For example, individuals ignored by their superiors may fear that leaving will either forego their chance to obtain a reference or portray them negatively should prospective employers seek referral information. In terms of external social support, we argued that the provision of available resources ought to lessen the sacrifice that an individual would endure by leaving an organization, thus heightening one’s intention to quit following an ostracism episode. Although external social support was found to be an important moderator of the relationship between ostracism and quitting intentions, the direction of this moderation differed between the two studies with high support weakening this relationship in Study 1 and strengthening it in Study 2. So why would both studies replicate findings for citizenship and deviance behaviors, but not turnover intentions?

One possible explanation might be related to the nature of the intention to quit as an outcome variable. Although turnover represents the final stage of withdrawal from an organization, recent work suggests that its intention can be a precursor to both prosocial and deviant behaviors (Mai et al., 2016). Turnover intentions reduce helping and increase deviance by reducing the relational and increasing the transactional aspects, respectively, of the psychological contract between the employee and the organization (Mai et al., 2016). If this is the case, then affect should have a greater influence on the proximal turnover intention than the more distal behaviors of helping and harming. In addition, a recent meta-analysis showed that, among different types of rejection, imagined rejection was most likely to elicit negative affect with an effect size twice as large as that of relived or directly experienced rejection (Blackhart et al., 2009). The weaker emotional impact associated with reporting actual ostracism in Study 2 might enable one to apply a more calculated approach when determining whether or not to quit given the available source of external social support. When negative affect is elicited by ostracism, as is the case of imagined ostracism in Study 1, victims will be more likely to use the existence of an external social support group as a buffer to thwarted belongingness needs instead of evaluating rationally whether their support will come to their aid during this transition. Consequently, such a reaction would help mitigate the effect of ostracism on quitting intentions. The difference in the affective impact of experienced versus imagined exclusion might not have been strong enough to affect the more distal and more concrete pro- and antisocial behaviors. However, given that we did not measure affect, the rationale we present for the divergent findings surrounding turnover intention remains speculative and should be the focus of future empirical work. The fact that different types of ostracism, i.e., imagined versus experienced in this case, have varied effects on some outcomes should urge researchers to employ disparate methodologies to test hypotheses within the same study and, more broadly, to conduct studies that attempt to replicate findings using different experimentally based ostracism paradigms.

More generally, our research speaks to the importance of educating managers and leaders about the detrimental effects of workplace ostracism. One implication is that organizations should consider providing a confidential means of reporting negative interpersonal workplace behaviors, including ostracism. Research has shown that organizations fostering a culture that underscores inclusiveness and communication transparency improves employees’ trust in the employer (Whitener et al., 1998).

### Limitations and Future Research

As with any research, our studies are not without limitations. First, in an effort to control for ostracizer gender in Study 1, we selected the male name ‘Edward’ rather than a neutral one like ‘Alex.’ Responses therefore did not include cases in which participants imagined that a female excluded them. Given the complexity of gender interactions, and despite research showing negative effects of ostracism perpetrated by a myriad of sources ranging from non-humans (e.g., computer programs; Zadro et al., 2004) to despised groups (e.g., the Ku Klux Klan; Gonsalkorale and Williams, 2007), the effect of ostracizer gender on outcomes of social exclusion is an avenue worth pursuing for future research. Women tend to be more person-oriented than males, with female leaders displaying more communal behaviors (e.g., empathy) than their agentic male counterparts (e.g., dominance) (Eagly, 1987; Vongas and Al Hajj, 2015b). Being ostracized by a woman may be interpreted differently than being ostracized by a man by virtue of the normative gender roles in Western society. Recent years have witnessed a gender role convergence in North America (Moen, 2003), and the potential moderating effect of ostracizer gender could be diminished in such a context. Although we replicated many of the findings across both studies in which ostracizer gender was not controlled, we can only presume at this juncture about the differential effects of ostracizer gender on other behaviors and intentions.

Second, we used participants from the United States and Canada, countries whose cultures resemble one another in many ways. One reason why ostracizees reduced their OCB-I may be related to the cultural dimension of temporal orientation. The social dilemma perspective views prosocial behaviors in the aftermath of ostracism as a long-term investment that a victim makes to secure reinclusion. In a recent paper, Balliet and Ferris (2013) demonstrated that a temporal orientation favoring short-term outcomes reduces post-exclusion prosocial behaviors whereas one favoring long-term outcomes increases such behaviors. Although their study treated temporal orientation as an individual-level characteristic, it has a history of scholarship at the country level. Defined as a “choice of focus for people’s efforts, be it the future, the present, or the past” (Hofstede, 2011, p. 8), temporal orientation in both the United States and Canada tends toward the short term. The fact that both our samples originated from transitory temporally oriented countries might explain why ostracism led to reductions in OCB-I. Ostracizees with a short-term orientation seek instant as opposed to delayed gratification making an investment in OCB-I unsuitable as a method of future positive returns. Our findings could be explained as ostracizees who attempt to seek redress against ostracizers by reducing their enactment of prosocial behaviors. Given that ostracism is qualitatively different from outright uncivil behaviors (e.g., supervisory abuse) when it comes to strength and clarity of intent (Ferris et al., 2013), we believe that an explanation that goes beyond simple social exchange is warranted. If social exchange alone is what motivates the extent of an ostracizee’s helping behaviors, then findings about the relationship between ostracism and OCB should be less contradictory than what the literature presents. Cultural differences might be an additional boundary condition that is contributing to the inconsistency in these findings, and future research using a different sample might reveal that responses to ostracism vary greatly across cultures. In fact, cultural dimensions other than temporal orientation could also play a role in how individuals respond to being excluded. Individuals from cultures characterized by a high power distance might assign more gravity to their supervisors’ opinions than those from a low-power distance culture, exaggerating the moderating effect of ostracizer status on the ostracism-outcome relationship. Collectivism, on the other hand, might buffer the observed negative and positive effects of ostracism on prosocial and deviant behaviors, respectively, due to the heightened commitment to the group and the existence of ample internal and external sources of social support. In fact, in a recent review of ostracism and incivility at work, Ferris et al. (2017) discuss the lack of comparative studies that examine the effects of culture on ostracism outcomes.

Third, an important limitation stems from our inability to establish a causal link between ostracism and our dependent variables, despite the use of an empirically based choice of controls for Study 2 and a time lag of 1 week for Studies 1 and 2. While our results are consistent with current theory (e.g., Williams, 2007; Ferris et al., 2008), threats to internal validity may have compromised assurance in the proposed relationships. For example, ostracizees may have reconciled with the ostracizer between the two times at which testing took place. Such an event or history as it is termed among research methodologists (Pedhazur and Schmelkin, 1991) would have undermined the outcomes observed here. As such, we caution researchers to be sensitive to contextual events in a given ostracism episode and to address other potential validity threats should they employ more prolonged longitudinal designs that assess variations in both independent and dependent variables. Examples of such threats include changes in participants’ learning or motivation (maturation) and the differential attrition of participants in the ‘ostracism’ versus ‘included’ treatments (mortality) (see also Campbell and Stanley, 1966).

Finally, although we use power differential as a possible mechanism through which ostracism affects citizenship, deviance, and turnover intention, we did not measure changes in people’s perceived level of power. One issue with measuring power both before and after an ostracism incident is that one needs to know (or control for) when ostracism takes place. In the field, this might be challenging unless one employs an experience sampling method (e.g., Fraley and Hudson, 2014) where measures would be separated by short intervals to capture times at which events and changes in power occur. In the laboratory, one could assess participants’ power, expose them to ostracism, and then measure their power again. We opted not to do so in the vignette study because measuring power before the ostracism description might have had unexpected effects on how respondents perceived the manipulation. Moreover, the idea of measuring power twice within such a short time period would most certainly have engendered carryover effects.

Two notable strengths of our work are that it investigated ostracism through a novel perspective, namely that of power, and used different methodologies to replicate findings. We therefore recommend researchers interested in ostracism to explore the nature of power relations between interacting parties using longitudinal or multiphased experimental designs. For those wishing to pursue additional vignette studies, organizational research is now making use of virtual reality technology that allows for improved realism (e.g., video; see Sauer, 2011). Workplace ostracism is a very common experience, with studies showing that over 70% of workers report being targets of exclusion in the recent past (O’Reilly et al., 2015). Moreover, it has detrimental consequences for victims despite assumptions claiming it to be a benign form of mistreatment (Robinson et al., 2013). As we have shown, ostracism also has serious adverse effects on organizations and, given its frequency and impact, should compel researchers to continue unearthing the factors that shape its outcomes.

## Ethics Statement

This study was carried out in accordance with the recommendations of the Canadian Tri-Council Policy Statement. All subjects gave written informed consent in accordance with the Helsinki Declaration of 1802. The protocol was approved by the Human Research Ethics Committee of Concordia University.

## Author Contributions

Conception, design, and acquisition of study data: JF. Analysis and/or interpretation of data: JF, RA, and JGV. Drafting and revising the manuscript for important intellectual content: JF, RA, and JGV. Approval of final version of manuscript: JF, RA, and JGV. Agreement to be accountable for all aspects of the work: JF, RA, and JGV.

## Conflict of Interest Statement

The authors declare that the research was conducted in the absence of any commercial or financial relationships that could be construed as a potential conflict of interest.
